# Supplementary material for: MiR-630 Promotes Radioresistance by Induction of Anti-Apoptotic Effect via Nrf2–GPX2 Molecular Axis in Head–Neck Cancer
Source: Cells. 2023 Dec 17;12(24):2853. doi: 10.3390/cells12242853 (PMC10741482; doi:10.3390/cells12242853)
Supplement: Supplementary file 1 [file cells-12-02853-s001.zip › miR-630-Figure S1.pdf]

Supplementary Material:

MiR-630 Promotes Radioresistance by Induction of Anti-Apoptotic Effect via Nrf2-–GPX2 Molecular Axis in Head-–Neck Cancer

Guo-Rung You, Ann-Joy Cheng, Eric Yi-Liang Shen, Kang-Hsing Fan, Yi-Fang Huang, Yu-Chen Huang, Kai-Ping Chang, and Joseph T. Chang

Figure S1

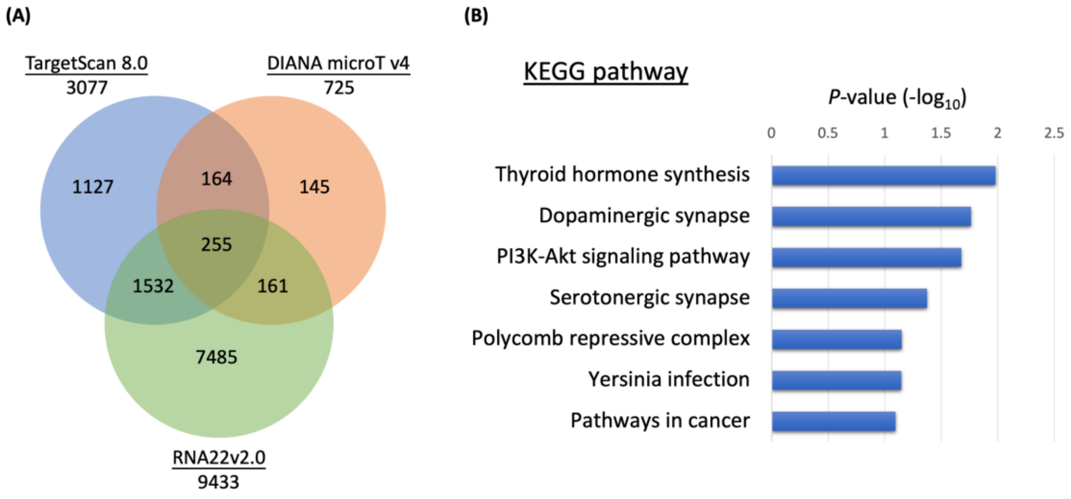

(C)

KEGG pathway	Gene list	Count
Thyroid hormone synthesis	GNAQ, CREB5, GPX8, HSPA5, SLC26A4	5
Dopaminergic synapse	GNAQ, GNG2, CREB5, MAOB, PPP2R5E, SCN1A	6
PI3K-Akt signaling pathway	BCL2L11, GNG2, PHLPP2, CREB5, PTEN, PRLR, PKN2, PPP2R5E, THBS2, TLR4	10
Serotonergic synapse	GNAQ, GNG2, MAOB, KNCD2, TPH1	5

(D)

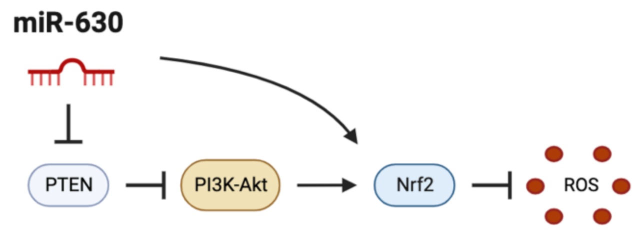

Figure S1. Prediction of PTEN as a target of miRNA-630. (A) Bioinformatics approach for predicting miR-630 targets involved the utilization of TargetScan 8.0 (3077 genes), DIANA-microT v4 (725 genes), and RNA22 v2 (9433 genes) prediction algorithms (all accessed on 25 November 2023). (B) Enrichment pathway analysis of the 255 common predicted target genes was conducted using the KEGG pathway via the DAVID online tool (Database for Annotation, Visualization, and Integrated Discovery, accessed on 25 November 2023). The significant pathways ($p \leq 0.05$) included thyroid hormone synthesis, dopaminergic synapse, PI3K-Akt signaling pathway, and serotonergic synapse. (C) The predicted target list of these prominent pathways was listed in a table. (D) A schematic representation hypothesizes that miR-630 may modulate cellular ROS levels by inhibiting the PTEN tumor suppressor and via the PTEN/PI3−Akt/Nrf2 molecular axis.
