# Supplementary material for: MiR-630 Promotes Radioresistance by Induction of Anti-Apoptotic Effect via Nrf2–GPX2 Molecular Axis in Head–Neck Cancer
Source: Cells. 2023 Dec 17;12(24):2853. doi: 10.3390/cells12242853 (PMC10741482; doi:10.3390/cells12242853)
Supplement: Supplementary file 1 [file cells-12-02853-s001.zip › miR-630-Table S1.pdf]

Table S1. Clinical characteristics of patients recruited in this study.

Item	Cancer	Normal
**Gender**		
Male	62 (86%)	30 (88%)
Female	10 (14%)	4 (12%)
**Age**		
Range (year old)	32-80	23-80
Mean ± SD (years)	52.6 ± 10.3	50.8 ± 12.5
≤ 40 years old	9 (13%)	6 (18%)
41-50 years old	20 (28%)	9 (26%)
51-60 years old	26 (36%)	12 (35%)
≥ 61years old	17 (23%)	7 (21%)
**Habits**		
Alcohol drinking	24 (33%)	
Betel quid chewing	37 (51%)	
Cigarette smoking	44 (61%)	
**Pathologic T-status**		
T1-T2	44 (61%)	
T3-T4	28 (39%)	
**Pathologic N-status**		
pN 0	29 (40%)	
pN+	43 (60%)	
**Pathologic overall stage**		
I - II	28 (39%)	
III - IV	44 (61%)	
**Total**	72 (100%)	34 (100%)
