# Supplementary material for: MiR-630 Promotes Radioresistance by Induction of Anti-Apoptotic Effect via Nrf2–GPX2 Molecular Axis in Head–Neck Cancer
Source: Cells. 2023 Dec 17;12(24):2853. doi: 10.3390/cells12242853 (PMC10741482; doi:10.3390/cells12242853)
Supplement: Supplementary file 1 [file cells-12-02853-s001.zip › miR-630-Table S2.pdf]

Table S2. List of the primers used in the present study.

Gene	Primer Sequences (5′ -> 3′)
GCLC	F: AACTCAGACATTGGATGGAG
	R: ACCTTTGACAGTGGAATGAG
GCLM	F: AGGGAGTTTCCAGATGTCTT
	R: AAGCAATGATCACAGAATCC
GPX2	F: TCATTGCCAAGTCCTTCTAT
	R: TGTTCAGGATCTCCTCATTC
MnSOD	F: CAAAGGGGAGTTGCTGGAAG
	R: AGCAGTGGAATAAGGCCTGT
	F: GGAGCGAGATCCCTCCAAAAT
GAPDH	R: GGCTGTTGTCATACTTCTCATGG
