# Supplementary material for: MiR-630 Promotes Radioresistance by Induction of Anti-Apoptotic Effect via Nrf2–GPX2 Molecular Axis in Head–Neck Cancer
Source: Cells. 2023 Dec 17;12(24):2853. doi: 10.3390/cells12242853 (PMC10741482; doi:10.3390/cells12242853)
Supplement: Supplementary file 1 [file cells-12-02853-s001.zip › miR-630-Table S3.pdf]

Table S3. List of the antibodies used in the present study.

Antibody	Catalog number (Company)
Bak	#12105 (Cell Signaling Technology Danvers, MA, USA)
Bax	#5023 (Cell Signaling Technology Danvers, MA, USA)
β-actin	NB600-501 (Novus, Centennial, CO, USA)
C-Caspase-3	#9664 (Cell Signaling Technology Danvers, MA, USA)
P-Caspase-3	#14220 (Cell Signaling Technology Danvers, MA, USA)
C-Caspase-7	#8438 (Cell Signaling Technology Danvers, MA, USA)
P-Caspase-7	#12827 (Cell Signaling Technology Danvers, MA, USA)
C-Caspase-9	#52873 (Cell Signaling Technology Danvers, MA, USA)
P-Caspase-9	#9508 (Cell Signaling Technology Danvers, MA, USA)
γ-H2AX	ab2893 (Abcam, Cambridge, UK)
Nrf2	#12721 (Cell Signaling Technology Danvers, MA, USA)
GPX2	ab137431 (Abcam, Cambridge, UK)
Anti-rabbit IgG-HRP	#7074 (Cell Signaling Technology Danvers, MA, USA)
Anti-mouse IgG-HRP	#7076 (Cell Signaling Technology Danvers, MA, USA)
Anti-rabbit IgG- Alexa Fluor® 488	A21206 (Invitrogen, Carlsbad, CA, USA)
